# Adherence to antihypertensive fixed-dose combination among Egyptian patients presenting with essential hypertension

**DOI:** 10.1186/s43044-020-00044-6

**Published:** 2020-03-05

**Authors:** Mahmoud Hassanein

**Affiliations:** Cardiology Department, Alexandria University, Alexandria, Egypt

**Keywords:** Medication adherence, Hypertension, MMAS-8

## Abstract

**Background:**

Many patients with hypertension require more than one drug to achieve blood pressure control. They are prescribed with fixed-dose combination (FDC) antihypertensive therapy rather than monotherapies. Although it is commonly admitted that the use of FDC may improve compliance to treatment, adherence rates in patients receiving FDCs have not been documented. Therefore, the aim of this study was to assess the adherence to treatment in patients receiving FDCs of antihypertensive medications in a real-world setting in Egypt.

**Results:**

We conducted a multi-center cross-sectional study over a period of 1 year from Jan 2017 to Jan 2018. We included patients above 21 years old with essential hypertension who were already prescribed with an FDC of antihypertensive treatment for at least 3-month duration. We assessed the adherence to treatment by patient self-assessment using the Morisky 8-Item Medication Adherence Scale (MMAS 8).

This study enrolled 2000 hypertensive Egyptian patients. The mean age of enrolled patients was 55.8 ± 10.9 years. Male to female ratio was 1.08. The mean MMAS score was 6.5 ± 1.9. Our analysis showed that 825 (41.3%) patients reached high adherence score, 523 (26.2%) medium adherence, and 652 (32.6%) low adherence.

Furthermore, Male patients showed higher adherence rate than females (56.4% versus 43.6%, *p* < 0.001). Out of 746 patients with controlled blood pressure (< 140/90), 387 (51.9%) patients were highly adherent to treatment. Higher level of education was significantly associated with high adherence rate; 559 (67.8%) patients were university graduates, 232 (28.1%) had primary/secondary school education, and 34 (4.1%) were illiterate (*p* < 0.001).

Moreover, once daily (99.2%) fixed-dose combination was associated with higher adherence rate than twice regimen daily (0.8%), *p* = 0.03. Multivariate logistic regression analysis showed that patients with high level of education, employed patients, and patients with controlled blood pressure have high adherence rate to medication.

**Conclusions:**

Higher adherence to medication is associated with high level of education and employment, and it can lead to better blood pressure control. Thus, patient education programs may increase patients’ adherence to their medication.

## Background

Hypertension (HTN) is a major global public health concern and is the leading cause of global death or disability [1]. About 30–40% of the adult population in the developed world suffer from this condition [2]. Despite the availability of many safe, effective, and tolerable medications, the treatment of HTN remains suboptimal [3]. In Egypt, HTN is highly prevalent, and rates of treatment, control, and awareness are relatively low [4]. It was estimated that the prevalence of hypertensive patients among Egyptian adults (≥ 25 years old) was 26.3%. In 60% of patients, HTN is complicated by the presence of other cardiovascular risk factors, leading to increased cardiovascular morbidity and mortality [5]. About 37% of hypertensive patients are receiving monotherapy. The 2018 European Society of Cardiology (ESC) and the European Society of Hypertension (ESH) guidelines reported that the following drugs demonstrated effective reduction of blood pressure (BP) and cardiovascular events (CV): calcium channel blockers (CCBs), beta-blockers, angiotensin-converting enzyme (ACE) inhibitors, angiotensin II receptor blockers (ARBs), and diuretics [6]. They also recommended combination therapy (ACE inhibitor or an ARB with a CCB or diuretic) as initial therapy for most hypertensive patients [7]. Moreover, they stated that patients should be subjected to a three-drug combination if BP not controlled on a two-drug combination [8].

Target BP control in hypertensive patients cannot be achieved without “good” adherence to prescribed medications according to healthcare providers’ instructions [9]. According to the observational evidence, the most important causes of poor BP control are the poor adherence to treatment and the lack of therapeutic action, which may correlate with a high risk of CV events [10–12]. Poor adherence can be defined as suboptimal daily use of prescribed regimens or early discontinuation of treatment [13, 14]. It was reported that after 6 months, more than one third of patients stop their initial treatment. This ratio may increase to half of the hypertensive patients after 1 year [15]. Therefore, the simplification of the treatment strategy and development of new approaches for the management of hypertension is a priority to improve the adherence to treatment and BP control [16]. In this study, we aimed to assess the adherence of the fixed-dose combinations (FDC) of available antihypertensive medications in a real-world sample in Egypt by patient self-assessment using the Morisky 8-Item Medication Adherence Scale (MMAS 8) [17].

## Methods

We followed Strengthening the Reporting of Observational Studies in Epidemiology (STROBE) statement guidelines when reporting this manuscript. This study was approved by the Egyptian Ministry of Health.

### Study design, study setting, and study participants

This was a multi-center, cross-sectional, observational study that was conducted over a period of 1 year from Jan 2017 to Jan 2018. We included 2000 Egyptian hypertensive patients from 100 centers spanning the Mediterranean coast, Nile Delta region, and southern Egypt.

We included patients meeting the following criteria: (1) males or females aged > 21 years; (2) patients with essential hypertension who were prescribed antihypertensive treatment with FDC for at least 3-month duration. All patients signed informed consent; and (3) patients willing to give written informed consent. We excluded all patients with severe renal impairment (GFR < 30 ml/min) or have any one of the following conditions: pregnancy, lactation, secondary hypertension, hypersensitivity to the used medications, or participating in other clinical studies.

### Study assessments and data collection

For each eligible patient, we reported the following data; during the recruitment process, the following data were collected for each patient eligible for the study: socio-demographic characteristics (age, sex, ethnicity, weight, and height), educational status, marital status, and occupation. Furthermore, we collected the data regarding the duration of hypertension, number of previous antihypertensive medications, duration, and type of treatment with FDC of anti-hypertension medications. Moreover, data regarding blood pressure measurements, medical history/comorbidity (e.g., smoking, diabetes, dyslipidemia, peripheral arterial disease, cardiovascular disease, congestive heart disease, and chronic pulmonary disease), family history of hypertension and cardiovascular disease, and self-administrated MMAS 8 questionnaire were obtained. Highly adherent patients were identified with the score of 8 on the scale, medium adherers with a score of 6 to < 8, and low adherers with a score of < 6.

All investigators received a formal, standardized training for blood pressure measurements. The blood pressure was assessed in the sitting position following a 5-min rest using a standardized automated sphygmomanometer (Omron T9P). A total of three readings were obtained, in which the first reading was discarded and the average of the second and third readings was obtained. Data were collected in the form of case report form (CRF). The CRFs were computerized with the data validation process, in which any raised inconsistencies led to appropriate queries to investigator resolution who is obliged to respond by confirming or modifying the inconsistent data. All of the key data were checked for each CRF (absence of missing data and consistency control).

### Study objectives

The primary objective was to measure the percentage of patients highly adherent to the treatment with FDC of anti-hypertension medications. The secondary objectives were to identify different patients’ profiles by comparing the characteristics of patients with a high, medium, and low adherence to the medication and to determine the causes of poor adherence to medication.

### Sample size calculation

Assuming that high adherence to treatment would be achieved in 50% of patients, with ≤ 10% of patients non-evaluable, enrollment of 2000 patients would provide a precision of 2.3% in the calculation of the 95% CI. If the rate of 0adherence were to be either higher or lower than 50%, precision would be better.

### Statistical analysis

All categorical variables were presented in frequency and percentage, whereas numerical variables were presented with mean and SD. Categorical data were described by frequency and percentage. Comparative analysis and inferential statistics were performed using paired *t* test, Wilcoxon signed-rank, and McNamara test for continuous variables. Chi-square test was used in case of categorical variables. For all statistical tests, *P* value ≤ 0.05 was considered statistically significant and the 95% CI for the percentage of highly adherent patients has been calculated. Univariate and multivariate logistic regression analyses were applied to identify the predictive factors for achieving high adherence to antihypertensive treatment. Univariate logistic regression considered each factor individually and multivariate considered all factors simultaneously. Multivariate model selection was carried out using stepwise and forward method by removing variables from the model that were not significant. The Hosmer–Lemershow test was used to measure the goodness-of-fit of the logistic regression model with *p* value ≤ 0.05 indicating poor fitness of model. Odds ratios (OR) with 95% confidence intervals (95% CI) were provided. All statistical tests were performed using SPSS program version 25.

## Results

### Baseline characteristics and demographic analysis

The mean age of enrolled patients was 55.8± 10.9 years old. The majority of participants were males 1040 (52.0%). Regarding the physical activity, 451 (22.6%) were inactive, 676 (33.8%) with low activity, 667 (33.4%) with moderate activity, and 206 (10.3%) with high activity. About two thirds of patients 1503 (75.1%) never smoke, 299 (15.0%) smokers, and 198 (9.9%) quit smoking. All demographics data are presented in Table 1.

**Table 1:** Socio-demographic characteristics of included patients

Parameters		Patients
Age, year		55.8 ± 10.9
Gender	Male	1040 (52%)
	Female	960 (48%
Height, cm		169.1 ± 8.3
Weight, Kg		89.5 ± 16.2
Body mass index, Kg/m^2^		31.4 ± 5.9
Blood pressure control	SBP < 140 mmHg	847 (42.4%)
	DBP < 90 mmHg	1109 (55.5%)
	BP < 140/90 mmHg	746 (37.3%)
Ethnicity	Middle eastern	1665 (83.3%)
	White/caucasian	300 (15%)
	Black	35 (1.8%)
Educational level	College/university	1160 (58%)
	Primary/secondary school	669 (33.5%)
	Illiterate	171 (8.6%)
Employment status	Full time	860 (43%)
	Retired	355 (17.8%)
	Housekeeper	336 (16.8%)
	Not employed	246 (12.3%)
	Part time	203 (10.2%)
Marital status	Married	1616 (80.8%)
	Widowed	248 (12.4%)
	Divorced	71 (3.6%)
	Single	65 (3.3%)
Patient daily physical activity/exercise level	Inactive	451 (22.6%)
	Low	676 (33.8%)
	Moderate	667 (33.4%)
	High	206 (10.3%)
Patient have any diet control/regimen for hypertension	Yes	641 (32.15%)
	No	1359 (68%)
Smoking status	Never	1503 (75.1%)
	Currently	299 (15%)
	Quit	198 (9.9%)

Data were presented as mean± SD for continuous data and frequency (%) for categorical variables Percentages. *SBP*; systolic blood pressure, *DBP*; diastolic blood pressure, *BP*; blood pressure

The mean systolic blood pressure (SBP) was 140.5 ± 16.8 mmHg, and the diastolic blood pressure (DBP) was 86.1 ± 10.0 mmHg. Controlled SBP (< 140 mmHg) was presented in 847 (42.4%) patients. In addition, the mean heart rate (HR) was 79 ± 8.8 beat/min. The mean duration since diagnosis of hypertension was 9.1 ± 7.3 years. Interestingly, 1181 (59.1%) patients have a family history of hypertension, and 461 (23.1%) have no family history of hypertension.

### Previous antihypertensive medications

Concerning antihypertensive medication, 602 (30.1%) patients received monotherapy, 606 (30.3%) patients received two types of antihypertensive drugs, 326 (16.3%) patients received triple combination therapy, and 145 (7.2%) patients received four types of antihypertensive drugs. The mean duration of treatment with FDC of anti-hypertension medications was 39.2 ± 36.9 months. The most frequent combination used was ARBs/HCTZ 846 (42.3%), then 337 (16.9%) ACE-I/HCTZ, 251 (12.6%) ARBs/CCB, and 227 (11.4%) triple combinations (ARBs/CCB/HCTZ). More than 98% of patients received FDC once time per day.

### Medical history and comorbidity

The most frequent comorbidities were dyslipidemia 987 (49.35%), diabetes mellitus 885 (44.25%), obesity 566 (28.3%), and coronary artery disease 309 (15.45%). We summarized the medical and surgical history of patients according to the Medical Dictionary for Regulatory Activities (MedDRA) and the concomitant medications in *S*upplementary file 1 and 2*.*

### Adherence to medication by MMAS

The mean value of adherence to medication by MMAS was 6.5 ± 1.9. Our analysis showed that 41.3% (95% CI [39.1 to 43.4%]) patients reached the high adherence, 26.2% (95% CI [24.3 to 28.1%]) of patients were medium adherent, and 32.6% (95% CI [30.6 to 34.7%]) of the patients were low adherent to the treatment. The correlation between the medication adherence and patient’s demographics is shown in Table 2 and Table 3.

**Table 2:** Adherence to the medication comparison with the categorical baseline parameters

Parameters		High (*n* = 825)	Medium (*n* = 523)	Low (*n* = 652)	*P* value
Gender	Male	465 (56.4%)	277 (53%)	298 (45.7%)	<0.001
	Female	360 (43.6%)	246 (47%)	354 (54.3%)	
Ethnicity	Middle eastern	717 (86.9%)	405 (77.4%)	543 (83.3%)	<0.001
	White/caucasian	100 (12.1%)	106 (20.3%)	94 (14.4%)	
	Black	8 (1%)	12 (2.3%)	15 (2.3%)	
Educational level	College/university	559 (67.8%)	310 (59.3%)	291 (44.6%)	<0.001
	Primary/secondary school	232 (28.1%)	164 (31.4%)	273 (41.9%)	
	Illiterate	34 (4.1%)	49 (9.4%)	88 (13.5%)	
Marital status	Married	678 (82.2%)	431 (82.4%)	507 (77.8%)	0.017
	Widowed	92 (11.2%)	51 (9.8%)	105 (16.1%)	
	Divorced	25 (3%)	22 (4.2%)	24 (3.7%)	
	Single	30 (3.6%)	19 (3.6%)	16 (2.5%)	
Employment status	Full time	423 (51.3%)	220 (42.1%)	217 (33.3%)	<0.001
	Retired	163 (19.8%)	85 (16.3%)	107 (16.4%)	
	Housekeeper	96 (11.6%)	74 (14.1%)	166 (25.5%)	
	Not employed	64 (7.8%)	77 (14.7%)	105 (16.1%)	
	Part time	79 (9.6%)	67 (12.8%)	57 (8.7%)	
Patient daily physical activity/exercise level	Inactive	160 (19.4%)	116 (22.2%)	175 (26.8%)	<0.001
	Low	266 (32.2%)	154 (29.4%)	256 (39.3%)	
	Moderate	307 (37.2%)	191 (36.5%)	169 (25.9%)	
	High	92 (11.2%)	62 (11.9%)	52 (8.0%)	
Patient have any diet control/regimen	No	523 (63.4%)	338 (64.6%)	498 (76.4%)	<0.001
	Yes	302 (36.6%)	185 (35.4%)	154 (23.6%)	
Smoking status	Never	646 (78.3%)	387 (74.0%)	470 (72.1%)	0.066
	Currently	108 (13.1%)	78 (14.9%)	113 (17.3%)	
	Quit	71 (8.6%)	58 (11.1%)	69 (10.6%)	
Family history of HTN	Yes	500 (71.5%)	294 (69.5%)	387 (74.4%)	0.236
	No	199 (28.5%)	129 (30.5%)	133 (25.6%)	
Frequency/day of last fixed-dose combination	Once	818 (99.2%)	513 (98.1%)	635 (97.4%)	0.031
	Twice	7 (0.8%)	10 (1.9%)	17 (2.6%)

**Table 3:** Adherence to the medication comparison with the continuous baseline parameters

Parameters	High (*n* = 825)	Medium (*n* = 523)	Low (*n* = 652)	*P* value
Age, year	55.6 ± 11.1	55.8 ± 10.5	56.0 ± 11.1	0.823
BMI, Kg/m2	30.4 ± 5.2	31.3 ± 5.7	32.7 ± 6.6	< 0.001
SBP, mmHg	137 ± 15.6	138.8 ± 15.7	145.9 ± 18.0	< 0.001
DBP, mmHg	84.4 ± 9.3	85.0 ± 9.5	89.1 ± 10.4	< 0.001

Male patients showed higher adherence rate than females (56.4% versus 43.6%, *p* < 0.001). Higher level of education was significantly associated with high adherence rate: 559 (67.8%) university level, 232 (28.1%) primary/secondary school, and 34 (4.1%) illiterate, (*p* < 0.001). In addition, once daily (99.2%) fixed-dose combination was associated with high adherence rate than twice daily (0.8%), *p* = 0.03. More than 82% of the high adherent patients were married, and 11.2% were widowed. There is no significant difference between the smokers or non-smokers regarding to the adherence to medication (*p* = 0.066).

**Fig. 1:**
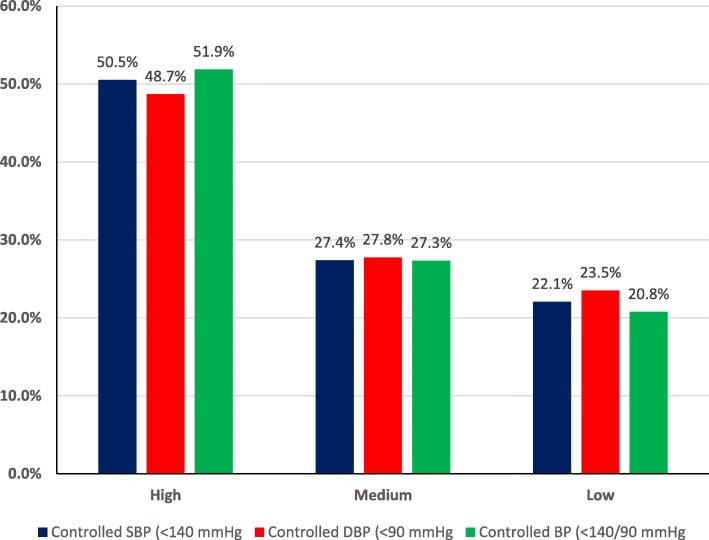
Adherence to the medication comparison with blood pressure control

Out of 847 patients with controlled SBP (< 140 mmHg), high adherence was found in 387 (51.9%) patients, medium adherence in 232 (27.4%) patients, and low adherence in 187 (22.1%) patients as presented in Fig. 1.

**Fig. 2:**
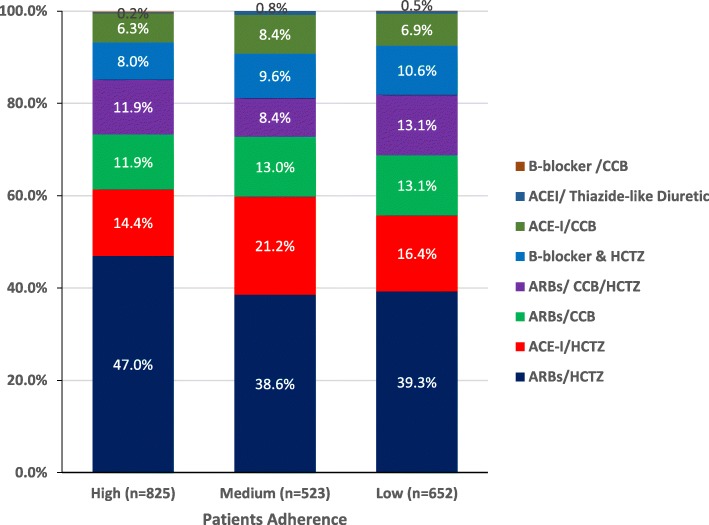
Adherence to the medication comparison with medication taken

Patients with high BMI, SBP, and DBP presented with low adherence to medications with statistically significant difference (*p* < 0.001). Also, patients with long history of hypertension showed lower adherence with medications (*p* = 0.795). In terms of patients with family history, 71.5% of high adherent patients are presented with a family history of hypertension. There is no significant difference between the scale degrees regarding the duration of treatment with FDC of anti-hypertension medications (*p* = 0.429). The majority of high adherent patients were receiving the combination of ARBs/HCTZ (47.0%) and ACE-I/HCTZ (14.4%, Fig. 2). Multivariate logistic regression analysis showed that patients with high level of education, employed patients, and patients with low SPB, DBP, and heart rate have high adherence rate to medication as seen in Table 4.

**Table 4:** Univariate and multivariate logistic regression analysis to identify the significant predictor variables for high adherence to medication

Parameters	Univariate analysis			Multivariate analysis		
	OR	95% CI for OR	*P* value	OR	95% CI for OR	*P* value
Age	1.007	0.994 to 1.021	0.277	-	-	-
Gender (ref: female)	1.016	0.781 to 1.322	0.906	-	-	-
Race (ref: non-middle eastern)	1.296	0.95 to 1.753	0.092	-	-	-
Education (ref: illiterate)						
Primary/secondary school	1.638	0.950 to 2.824	0.076	1.70	0.998 to 2.896	0.05*
College/university	2.191	1.270 to 3.778	0.005*	2.354	1.387 to 3.994	0.002*
Marital status (ref: not married)	0.840	0.622 to 1.133	0.253	-	-	-
Employment (ref: non employer)	1.681	1.258 to 2.247	< 0.001*	1.485	1.171 to 1.883	0.001*
Smoking (ref: smoker)	1.416	1.023 to 1.96	0.036*	-	-	-
BMI, Kg/m^2^	0.974	0.946 to 1.003	0.082	-	-	-
BMI (ref: > 30 Kg/m^2^)	1.102	0.793 to 1.531	0.562	-	-	-
SBP, mmHg	0.988	0.979 to 0.998	0.016*	0.989	0.980 to 0.998	0.021*
DBP, mmHg	0.983	0.967 to 0.999	0.033*	0.981	0.966 to 0.996	0.015*
HR, beat/min	0.969	0.955 to 0.983	< 0.001*	0.965	0.951 to 0.978	< 0.001*
Duration of HTN	1.004	0.987 to 1.022	0.61	-	-	-
Family history of HTN (ref: no family history)	0.918	0.717 to 1.174	0.493	-	-	-
Antihypertensive treatment (ref: ARBs/HCTZ)						
ACE-I/HCTZ	0.622	0.452 to 0.858	0.114			
ACE-I/CCB	0.998	0.652 to 1.528	0.993			
ARBs/CCB	0.806	0.567 to 1.145	0.229			
ARBs/CCB/HCTZ	0.773	0.526 to 1.136	0.190	-	-	-
β-blocker/HCTZ	0.732	0.496 to 1.082	0.118			
ACEI/thiazide-like diuretic	0.358	0.063 to 2.037	0.247			
β-blocker and CCB	1.335	0.178 to 10.008	0.779			

*statistically significant

## Discussion

In this cross-sectional study, we investigated the adherence of hypertensive patients to antihypertensive medications. Our findings showed that 41.3% of Egyptian hypertensive patients were highly adherent to antihypertensive medications. Interestingly, about 60% of patients have a family history with hypertension. The most frequent dual combinations were ARBs & HCTZ, ACE-I & HCTZ, ARBs & CCB; while, the most frequent triple combination is ARBs, CCB & HCTZ. Moreover, half of those patients have dyslipidemia, and 44.25% are suffering from diabetes mellitus. In terms of MMRS, 41.3% of patients had high adherence, 26.2% had medium adherence, and 32.6% had low adherence. These results are somewhat different from those of Tilea et al. [12] who reported better adherence rates : 69.8% of patients had high adherence, 20.3% had medium adherence, and 9.9% had low adherence through a prescription record review tool. However, some studies reported high levels of non-adherence to antihypertensive treatment. In a cross-sectional study performed in Ghana and Nigeria, 66.7% of patients were non-adherent to treatment. Non-adherence was related to age (younger patients) and education level [18]. In contrast, our results documented that high adherence rates were more often associated with male gender, middle eastern ethnicity, high level of education, full-time workers, and married persons. These findings are similar to those of Tilea et al. [12], Daniel and Veiga [19], Karakurt and Kaşikçi [20], and Tibebu et al [21].

Daniel and his colleagues found that adherence to therapy was better in the age group of 40–59 years and those > 80 years of age, and that the most highly adherent participants were 61–70 years old (31.4%). Moreover, 32 of these patients in the age group of 51–60 years (30.5%) had a moderate adherence, and half of their hypertensive patients who had low adherence rates (< 20%) to treatment were in the age group of 51–60 years, and, demonstrating significant (*p* = 0.0001) effects of age on treatment adherence [19]. Similarly, Bandi et al. reported that the higher prevalence of medication adherence was observed in older patients (34.0%) when compared to young patients (24.5%, *p* = 0.001). They explained this by the heavy alcohol consumption, lower education level, and lower knowledge of hypertension control in young patients [22].

Regarding the adherence difference in gender classification, we found that males showed highly significant adherence to treatment compared to females (56.4% versus 43.6%, *p* < 0.001). In contrast, Ambaw et al. reported that men were found to be less adherent when compared to women. They added that men are loaded by the outdoor activities which make them busy and make them forget their medications [23].

In this study, we found that highly educated and full-time employed patients showed high adherence to antihypertensive medications. In contrast, Lee et al. demonstrated that patients with primary or low education and patients who were unemployed or retired were more likely to be compliant [24]. This difference can be explained by the fact that medical service was relatively accessible in Hong Kong.

We found a significant difference between the MMAS degrees according to the number of previous antihypertensive medications; increased numbers of antihypertensive drugs are associated with intermediate and poor adherence (*p* = 0.019). This finding is similar to those of Mallat et al. [25] who found that increased numbers of antihypertensive drugs prescribed in the presence of comorbidities are associated with poor adherence. However, the available evidence is not yet sufficient to conclude that there are differences between an FDC and to free combination therapy in the management of HTN [26].

Treatment cost is one of the main driven factor of high non-adherence rates across all therapeutic areas, and the financial burden of the increasing cost of treatment can limit patients access to treatment, regardless of other determinants of socio-economic status [11, 27]. Previous reports showed that drug cost is accounted for a substantial proportion of drug non-adherence [28]. In the present study, there was no financial coverage of prescribed drugs which can limit our findings due to lack of standardization of this confounding factor.

Good doctor-patient communication, adequate knowledge, awareness of the disease and its possible complications, as well as the use of special containers for drug dispensing (blister packs, bottles, and timed dosing batches), remembering to renew the prescriptions, and then affording the price of the medication are important elements that affect the adherence to the treatment plan [20, 29, 30].

## Conclusion

In an Egyptian hypertensive population treated with FDCs, higher medication adherence rates were associated with higher education level, middle eastern ethnicity, employed patients, and lower BP levels. Patients with high adherence had lower BP levels and lower BMI as compared with patients with medium and low adherence. Developing awareness through patient education programs could increase patients’ adherence to their medication.

## Supplementary information

**Additional file 1:.** Medical History / Co-morbidity

**Additional file 2:.** Concomitant Medication

## Data Availability

The datasets generated during the current study are not publicly available but are available from the corresponding author on reasonable request.

## Abbreviations

ACE: Angiotensin-converting enzyme
ARBs: Angiotensin II receptor blockers
BP: Blood pressure
CCBs: Calcium channel blockers
CRF: Case report form
CV: Cardiovascular events
DBP: Diastolic blood pressure
ESC: European Society of Cardiology
FDC: Fixed-dose combination
HTN: Hypertension
MedDRA: Medical Dictionary for Regulatory Activities
MMAS 8: Morisky 8-Item Medication Adherence Scale
SBP: Systolic blood pressure
STROBE: Strengthening the Reporting of Observational Studies in Epidemiology
