# Supplementary material for: Adherence to antihypertensive fixed-dose combination among Egyptian patients presenting with essential hypertension
Source: Egypt Heart J. 2020 Mar 5;72:10. doi: 10.1186/s43044-020-00044-6 (PMC7058724; doi:10.1186/s43044-020-00044-6)
Supplement: Supplementary file 1 — Additional file 1:. Medical History / Co-morbidity [file 43044_2020_44_MOESM1_ESM.docx]

Supplementary File 1

| **Medical History / Co-morbidity** | **Count*** | **%** |
| --- | --- | --- |
| **MedDRA Coding (PT)** |  |  |
| Diabetes mellitus | 885 | 44.25 |
| Dyslipidaemia | 987 | 49.35 |
| Obesity | 566 | 28.3 |
| Coronary artery disease | 309 | 15.45 |
| Cerebrovascular disorder | 45 | 2.25 |
| Hypertensive heart disease | 42 | 2.1 |
| Cerebrovascular accident | 47 | 2.35 |
| Cardiovascular disorder | 56 | 2.8 |
| Nephropathy | 21 | 1.05 |
| Retinopathy | 10 | 0.5 |
| Chronic respiratory disease | 48 | 2.4 |
| Anaemia | 6 | 0.3 |
| Anxiety | 1 | 0.05 |
| Aortic stenosis | 1 | 0.05 |
| Aortic valve replacement | 3 | 0.15 |
| Arteriosclerosis | 1 | 0.05 |
| Asthma | 4 | 0.2 |
| Atrial fibrillation | 17 | 0.85 |
| Atrial septal defect repair | 1 | 0.05 |
| Atrioventricular block complete | 2 | 0.1 |
| Behcet's syndrome | 1 | 0.05 |
| Benign prostatic hyperplasia | 10 | 0.5 |
| Blood uric acid increased | 1 | 0.05 |
| Breast cancer | 5 | 0.25 |
| Breast cancer metastatic | 2 | 0.1 |
| Breast conserving surgery | 1 | 0.05 |
| Cancer surgery | 1 | 0.05 |
| Cholecystitis | 1 | 0.05 |
| Cholecystitis chronic | 4 | 0.2 |
| Cholelithiasis | 1 | 0.05 |
| Chronic gastritis | 2 | 0.1 |
| Chronic hepatitis C | 1 | 0.05 |
| Chronic sinusitis | 1 | 0.05 |
| Colitis ulcerative | 1 | 0.05 |
| Congestive cardiomyopathy | 1 | 0.05 |
| Coronary artery bypass | 1 | 0.05 |
| Deep vein thrombosis | 4 | 0.2 |
| Dementia | 1 | 0.05 |
| Depression | 4 | 0.2 |
| Diabetic nephropathy | 1 | 0.05 |
| Diabetic neuropathy | 2 | 0.1 |
| Dyslipidaemia | 1 | 0.05 |
| Echinococciasis | 1 | 0.05 |
| Erectile dysfunction | 1 | 0.05 |
| Factor V Leiden mutation | 1 | 0.05 |
| Gastritis | 9 | 0.45 |
| Gastritis erosive | 1 | 0.05 |
| Gastritis,Gastrooesophageal reflux disease | 1 | 0.05 |
| Gastrooesophageal reflux disease | 5 | 0.25 |
| Gout | 20 | 1 |
| Gouty arthritis | 5 | 0.25 |
| Guillain-Barre syndrome | 1 | 0.05 |
| Hepatic cirrhosis | 4 | 0.2 |
| Hepatic steatosis | 4 | 0.2 |
| Hepatitis C | 11 | 0.55 |
| Hyperthyroidism | 12 | 0.6 |
| Hypertrophic cardiomyopathy | 1 | 0.05 |
| Hyperuricaemia | 21 | 1.05 |
| Hypervitaminosis | 1 | 0.05 |
| Hypoglycaemia | 1 | 0.05 |
| Hypothyroidism | 30 | 1.5 |
| Hypouricaemia | 1 | 0.05 |
| Inflammatory bowel disease | 1 | 0.05 |
| Irritable bowel syndrome | 4 | 0.2 |
| Irritable bowel syndrome,Gastritis | 1 | 0.05 |
| Jaundice cholestatic | 1 | 0.05 |
| Left ventricular hypertrophy | 2 | 0.1 |
| Leukoderma | 1 | 0.05 |
| Ligament rupture | 1 | 0.05 |
| Liver disorder | 4 | 0.2 |
| Lung neoplasm malignant | 1 | 0.05 |
| Microalbuminuria | 1 | 0.05 |
| Migraine | 1 | 0.05 |
| Mitral valve replacement | 1 | 0.05 |
| Mitral valve replacement,Aortic valve replacement | 1 | 0.05 |
| Mitral valve replacement,Tricuspid valve repair | 1 | 0.05 |
| Myxoedema | 1 | 0.05 |
| Neuropathy peripheral | 1 | 0.05 |
| Non-alcoholic fatty liver | 2 | 0.1 |
| Osteoarthritis | 25 | 1.25 |
| Osteoarthritis,Osteoporosis | 1 | 0.05 |
| Osteodystrophy | 1 | 0.05 |
| Osteopenia | 1 | 0.05 |
| Osteoporosis | 2 | 0.1 |
| Otitis media | 1 | 0.05 |
| Pancreatic carcinoma | 1 | 0.05 |
| Parathyroid tumour malignant | 1 | 0.05 |
| Parkinsonism | 4 | 0.2 |
| Peripheral arterial occlusive disease | 1 | 0.05 |
| Peripheral vascular disorder | 1 | 0.05 |
| Prostatomegaly | 3 | 0.15 |
| Psoriasis | 1 | 0.05 |
| Pulmonary embolism | 3 | 0.15 |
| Renal transplant | 1 | 0.05 |
| Retinopathy | 1 | 0.05 |
| Retinopathy,Cataract | 1 | 0.05 |
| Rheumatic heart disease | 1 | 0.05 |
| Rheumatoid arthritis | 9 | 0.45 |
| Rhinitis allergic | 1 | 0.05 |
| Spinal osteoarthritis | 3 | 0.15 |
| Supraventricular tachycardia | 2 | 0.1 |
| Thalassaemia | 1 | 0.05 |
| Thrombocytosis | 2 | 0.1 |
| Thyroid cancer,Chemotherapy,radiotherapy | 1 | 0.05 |
| Thyroid disorder | 4 | 0.2 |
| Thyroidectomy | 1 | 0.05 |
| Toxic goitre | 1 | 0.05 |
| Transcatheter aortic valve implantation | 1 | 0.05 |
| Trigeminal neuralgia | 1 | 0.05 |
| Uterine leiomyoma | 1 | 0.05 |
| Varicose vein | 1 | 0.05 |
| Ventricular extrasystoles | 1 | 0.05 |
| Vertigo | 1 | 0.05 |
| Vitamin D deficiency | 1 | 0.05 |
| Wolff-Parkinson-White syndrome | 1 | 0.05 |
| Total | 3337 | 100 |
