# Supplementary material for: Adherence to antihypertensive fixed-dose combination among Egyptian patients presenting with essential hypertension
Source: Egypt Heart J. 2020 Mar 5;72:10. doi: 10.1186/s43044-020-00044-6 (PMC7058724; doi:10.1186/s43044-020-00044-6)
Supplement: Supplementary file 2 — Additional file 2:. Concomitant Medication [file 43044_2020_44_MOESM2_ESM.docx]

| **Concomitant Medication** | **Overall** | |
| --- | --- | --- |
|  | **Count*** | **%** |
| Analgesic | 7 | 0.35 |
| Anti-anginal | 199 | 9.95 |
| Anti-anxiety | 1 | 0.05 |
| Anti-arrhythmic | 32 | 1.6 |
| Anti-asthmatic | 38 | 1.9 |
| Anti-bone resorption | 1 | 0.05 |
| Anti-cancer | 3 | 0.15 |
| Anti-hyperthyroidism | 11 | 0.55 |
| Anti hyperuricemic | 4 | 0.2 |
| Anti-hypothyroidism | 43 | 2.15 |
| Anti-psoriasis | 1 | 0.05 |
| Anti-emetic | 2 | 0.1 |
| Anti-epileptic | 11 | 0.55 |
| Anti-erectile dysfunction | 1 | 0.05 |
| Anti-inflammatory | 28 | 1.4 |
| Anti-ischemic | 18 | 0.9 |
| Anti-obesity | 1 | 0.05 |
| Anti-oxidant | 3 | 0.15 |
| Anti-ulcer | 59 | 2.95 |
| Antibiotic | 15 | 0.75 |
| Anticholinergic | 1 | 0.05 |
| Anticoagulant | 44 | 2.2 |
| Antidepressant | 10 | 0.5 |
| Antidiabetic | 1292 | 64.6 |
| Antiflatulance | 2 | 0.1 |
| Antigout | 55 | 2.75 |
| Antihypertensive | 167 | 8.35 |
| Antimalarial | 1 | 0.05 |
| Antimicrobial | 1 | 0.05 |
| Antimuscarinic | 1 | 0.05 |
| Antineoplastic | 2 | 0.1 |
| Antiparkinson | 6 | 0.3 |
| Antiplatelets | 598 | 29.9 |
| Antipsychotic | 4 | 0.2 |
| Antirheumatic | 9 | 0.45 |
| Antispasmodic | 4 | 0.2 |
| Antithrombosis | 1 | 0.05 |
| Antitussive | 1 | 0.05 |
| Antivertigo | 11 | 0.55 |
| Antiviral | 2 | 0.1 |
| Anxiolytic | 2 | 0.1 |
| Benign Prostatic Hypertrophy Agent | 7 | 0.35 |
| Cardiotonic | 11 | 0.55 |
| Cerebral vasodilator | 11 | 0.55 |
| Contraceptive | 1 | 0.05 |
| Eye drops | 2 | 0.1 |
| Hepatic Protector | 6 | 0.3 |
| Hypochloremia | 1 | 0.05 |
| Hypolipidemic | 929 | 46.45 |
| Immunomodulating agents | 1 | 0.05 |
| Immunosuppressive drug | 4 | 0.2 |
| Laxatives | 2 | 0.1 |
| Liver supplement | 9 | 0.45 |
| Mucolytic agent | 3 | 0.15 |
| Muscle relaxant | 2 | 0.1 |
| Neuropathic pain agents | 2 | 0.1 |
| Peripheral vasodilator | 5 | 0.25 |
| Sobofoxate | 1 | 0.05 |
| Supplements and vitamines | 91 | 4.55 |
| Venotropic | 2 | 0.1 |
| Total | 3782 | 100 |
